# Spatial Autocorrelation Analysis of Chinese Inter-Provincial Industrial Chemical Oxygen Demand Discharge

**DOI:** 10.3390/ijerph9062031

**Published:** 2012-05-25

**Authors:** Xiaofeng Zhao, Xianjin Huang, Yibo Liu

**Affiliations:** 1 School of Government, Nanjing University, 22 Hankou Road, Nanjing 210093, China; Email: zhao-xf@126.com; 2 School of Geographic and Oceanographic Sciences, Nanjing University, 22 Hankou Road, Nanjing 210093, China; 3 International Institute for Earth System Sciences, Nanjing University, 22 Hankou Road, Nanjing 210093, China; Email: yiboliu@nwsuaf.edu.cn

**Keywords:** industrial COD discharge, spatial autocorrelation, Moran’s I, spatial pattern

## Abstract

A spatial autocorrelation analysis method is adopted to process the spatial dynamic change of industrial Chemical Oxygen Demand (COD) discharge in China over the past 15 years. Studies show that amount and intensity of industrial COD discharges are on a decrease, and the tendency is more remarkable for discharge intensity. There are large differences between inter-provincial discharge amount and intensity, and with different spatial differentiation features. Global spatial autocorrelation analysis reveals that Global Moran’s I of discharge amount and intensity is on the decrease. In space, there is an evolution from an agglomeration pattern to a discretization pattern. Local spatial autocorrelation analysis shows that the agglomeration area of industrial COD discharge amount and intensity varies greatly in space with time. Stringent environmental regulations and increased funding for environmental protections are the crucial factors to cut down industrial COD discharge amount and intensity.

## 1. Introduction

Chemical Oxygen Demand (COD), a specific comprehensive index to characterize the organic pollution in the environmental water sample, is mostly used as the important basis for determining the relative content of organic water pollutants in environmental monitoring and environmental impact assessments. Nutrients in industrial waste water and domestic wastewater discharged by human activity causes water eutrophication, algal bloom disasters, loss of ecological function and other problems. Due to its serious damaging effects, difficult treatment and slow restoration, it is treated as a global water pollution problem. Since industrial wastewater has complex chemical components, and is difficult to treat, degrade and purify, it is more harmful than domestic wastewater. Thus the control of COD discharge in the industrial wastewater is an important component in the prevention and control of water environmental pollution in China.

With the rapid economic development of society, since 2000, water eutrophication and blue-green algae have been frequent in such large-sized natural lakes as Taihu Lake, Chaohu Lake, Dianchi Lake, *etc*., however, in 1980s, those phenomena were mainly found in urban lakes in China. Waters adjacent to the Yangtze Estuary also fall into the frequent harmful red tide areas in China, where about 1/4 recorded red tides occur [1,2]. According to the examination result of 2008 Environmental Protection Special Action carried out by eight ministries and commissions under the State Council, there is a low attainment rate of water quality for 113 urban drinking water sources under key monitoring across China, and among 243 surface water sources, 159, accounting for 65%, have passed the attainment deadline, while the remaining 84 sources, accounting for 35%, fail [3]. It was revealed in the 2009 Water Environmental Quality Situation in China report issued by the Chinese Ministry of Environment Protection that surface water pollution is still serious in all China, and lakes (reservoirs) have prominent eutrophication problems. The Chinese government has adopted a strategy combining amount control and intensity control in the prevention and control of water pollution, which cannot only control the general level of COD discharge within the region, but also reflect the relation between COD discharge and economic development, thus realizing the wide use of this index in regional pollution monitoring and control as well as discharge control of industrial and enterprise pollution.

Water pollution and ecological environment degradation caused by COD discharge have aroused extensive concern around the World. Existing research mainly concentrates on COD determination [4,5,6,7,8] and treatment technologies [9,10,11,12]. Some documents have offered some analysis of temporal and spatial variation of COD. For example, researches on Lake Taihu [13,14,15], the coastal rivers of Western Finland [16], Yamuna River [17], urbanized rivers in Algeria [18], and other regions [19,20,21] reflect the distribution characteristics and variation tendency of COD on a small time scale and space scale. However, there is a relative lack of studies on the spatial characteristics of COD discharge over long time sequenced and a large spatial scale, especially the dependency and heterogeneity of COD spatial distribution. Moreover, owing to quite different economic development levels, degree of industrialization, water pollution treatment technology and investment in water environment control of the various provinces in China, there are great spatial differences regarding effective control of COD discharge and water environment. In geography, spatial autocorrelation analysis is an important method for the quantitative study of various problems involving spatial relations within natural, economic and social fields, as well as an effective measure to analyze spatial patterns. It has been widely used in multiple areas like ecology [22], nosography [23], sociology [24], forestry [25], biology [26], regional economy [27], land use [28], *etc.* Using a spatial autocorrelation analysis method, this study analyzes the spatial dynamics of industrial COD discharge intensity in China in the past 15 years and probes into its spatial heterogeneity and development rules. It provides a reference for industrial development strategies and relevant water environmental protection policies, a basis for environmental protection macro strategy, which is currently studied and implemented by the state, and a beneficial reference for the construction of an environment-friendly society and ecological civilization.

## 2. Materials and Methods

### 2.1. Data Sources

As referred in this study, two indexes have been adopted for the measurement of industrial COD discharge: amount and intensity. Industrial COD discharge amount refers to the quantity of COD contained in the industrial wastewater of the whole of China or each province; industrial COD discharge intensity refers to the industrial COD discharge amount *per* unit industrial added value, *i.e.*, industrial COD discharge amount divided by the industrial added value. Hence, this study involves environmental and economic data, which are respectively obtained from the China Environment Yearbook (1997–2011) and China Statistical Yearbook (1997–2011). To analyze the characteristics and variation rules of industrial COD discharge amount and intensity in different economic development stages, time is divided into “9th Five-Year” Period (1996–2000), “10th Five-Year” Period (2001–2005) and “11th Five-Year” Period (2006–2010) according to the Chinese economic and social development five-year plans. However, Chongqing Municipality was separated from Sichuan Province and established as a municipality directly under the Central Government in 1997. Thus its environmental and economic statistic data in 1996 have to be obtained from the Sichuan Province Environment Yearbook 1997 and Sichuan Province Statistical Yearbook 1997, which cannot be obtained currently. In addition, the China Environment Yearbook 2011 has not been published yet by the Ministry of Environment Protection, consequently relevant data of 2010 are not available either. For this reason, this study defines the time zone of analysis as 1997–2009. In addition, data of Hong Kong, Macao and Taiwan were not available either.

### 2.2. Analytic Methods

A three-step analysis process was followed: first the spatiotemporal distribution of the COD in industrial wastewater was introduced. Following this, the analysis focused on two aspects of the spatial clustering: global spatial autocorrelation and local spatial autocorrelation of industrial wastewater COD discharges.

In order to identify and measure the strength of spatial patterns, showing how the industrial wastewater COD discharge were correlated in these provinces, Moran’s *I* values was calculated and assessed by testing a null hypothesis. Rejection of the null hypothesis implies a non-random spatial pattern also referred to as spatial autocorrelation. In particular, spatial autocorrelation measures the nature and strength of interdependence between data. We speak of positive spatial autocorrelation where similar values tend to occupy adjacent locations, whereas negative autocorrelation implies that high values tend to be located next to low ones. On the other hand, if the spatial arrangement is completely random, then this implies absence of spatial autocorrelation. Moran’s *I* ranges approximately from +1 to −1. The closer Moran’s *I* tends to 1, the stronger the positive spatial autocorrelation is, while the closer Moran’s *I* tends to −1, the stronger the negative spatial autocorrelation is，and its expected value in the absence of autocorrelation approximates zero [29,30]. Neighbor relationships are typically expressed in a row-standardised spatial weights matrix “*w*” [31], the elements of which *w_ij_*correspond to the spatial weights assigned to pairs of units *i* and *j*. In the present analysis, neighbors were defined using rooks contiguity, which considers that all provinces with common borders are neighbors. The global Moran’s *I* is defined as Equation (1), and the local Moran’s *I* is defined as Equation (2) [32]:


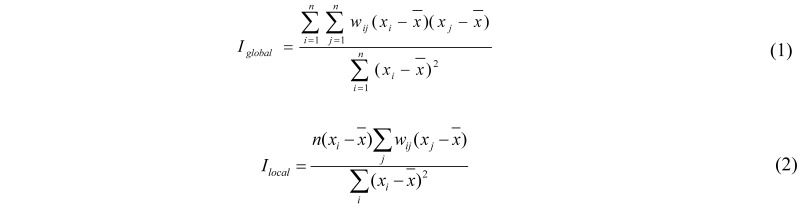


where *n* is the number of spatial units; *x_i_* and *x_j_* are the values of variable *x* in spatial unit *i* and *j*; 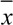 is the average over all spatial units of the variable. *w_ij_* is the spatial weight matrix that measures the strength of the relationship between two spatial units. 

Before calculating Moran’s *I*, standardization processing shall be conducted to observe data. To analyze and observe the spatial pattern of variations, test of significance is requited, to ensure the correctness of inferred conclusion based on a certain probability. Test of significance adopts a Z test (Equation (3)):


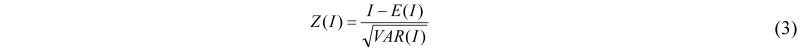


where Z(*I*) represents the significance level of Moran’ I, E(*I*) is the mathematic expectation of Moran’ *I*, and VAR(*I*) is variation.

The global Moran’s *I* does not indicate where the clusters are located or what type of spatial autocorrelation is occurring [33]. The local indicator of spatial autocorrelation (LISA) was therefore applied as an indicator of local spatial association. The LISA significance map was created incorporating information about the significance of the local spatial patterns. In particular, the map results in a spatial pattern consisting of five categories [33]: (i) “High-High” indicates higher values surrounded by neighboring units with higher values, and it means positive spatial autocorrelation, (ii) “Low-High” indicates low values adjacent to neighboring units with higher values, and it means negative spatial autocorrelation, (iii) “Low-Low” indicates lower values surrounded by neighboring units with lower values, and it means positive spatial autocorrelation, (iv) “High-Low” indicates higher values adjacent to neighboring units with lower values, and it means negative spatial autocorrelation, and (v) “Not Significant” indicates that there is no spatial autocorrelation. The high-high and low-low locations (positive local spatial autocorrelation) are typically referred to as spatial clusters, while the high-low and low-high locations (negative local spatial autocorrelation) are termed spatial outliers. It should be kept in mind that the so-called spatial clusters shown on the LISA cluster map only refer to the core of the cluster [34]. 

Both Moran’s *I*, including global Moran’s *I* and local Moran’s *I*, and LISA calculations are performed using GeoDa 0.9.5i [35], a spatial statistical freeware package developed by Luc Anselin and co-workers in the Department of Geography, University of Illinois, Urbana-Champaign, IL, USA. 

## 3. Results and Discussion

### 3.1. Evolution and Spatial Distribution of Industrial COD Discharge

**Figure 1:**
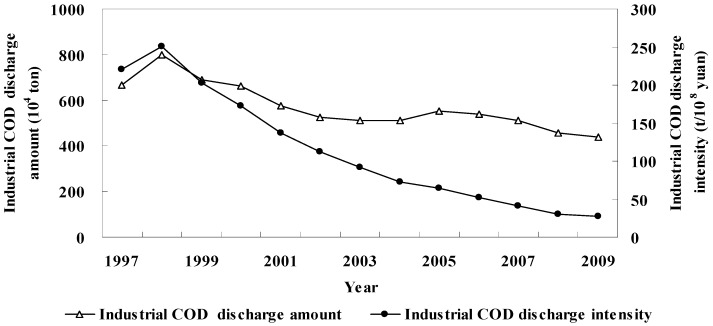
Industrial COD discharge amount and intensity from 1997 to 2009.

In the past 15 years, there was an obvious cut in industrial COD discharges in China. During the “9th Five-Year” period, it first increased, reaching a maximum value of 800.61 × 10^4^ tons in 1998, and then decreased. During the “10th Five-Year” period, there was little change, remaining at 500 × 10^4^ to 550 × 10^4^ tons. Up to the “11th Five-Year” period, it decreased slowly and progressively, down to a minimum value of 439.68 × 10^4^ tons in 2009. Though experiencing fluctuation in a narrow range, it still takes on a general overall decreasing trend (Figure 1).

Compared with the changing trend of industrial COD discharge gross amount, industrial COD discharge intensity experienced a more remarkable decreasing trend (Figure 1). It increased slightly in the earlier “9th Five-Year” Period, reached the maximum of 250.07 tons/hundred million Yuan in 1998, and later went down to 27.92 in 2009. This later considerable decrease was due to a combined reduction of industrial COD discharge amounts and a sharp increase in industrial output.

**Figure 2:**
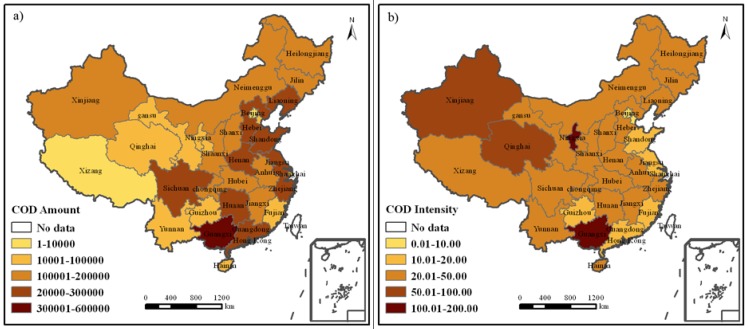
Industrial COD discharge amount and intensity in 2009. (**a**) Industrial COD discharge amount in 2009; (**b**) Industrial COD discharge intensity in 2009.

Inter-provincial industrial COD discharge amount varied very much (Figure 2a). Taking 2009 as an example, the COD discharge amount in the Guangxi Zhuang Autonomous Region was 51.88 × 10^4^ tons, far higher than that of other provinces; next was Henan Province, which was 29.77 × 10^4^ tons; and over 20 × 10^4 ^tons in such provinces like Shandong, Jiangsu, Sichuan, Zhejiang, Hebei, Liaoning, Guangdong and Hunan. Tibet Autonomous Region had the least industrial COD discharge amount of 736.85 tons; while Beijing was the second least of 4,898.1 tons. Industrial COD discharge amount was between 10 × 10^4^ and 20 × 10^4^ tons in places like Hainan, Guizhou, Tianjin, Shanghai, Qinghai, Gansu, Fujian, Yunnan, Ningxia, *etc.*, and between 20 × 10^4^ and 30 × 10^4 ^tons in other places.

The degree of industrialization and industrial COD discharge amount vary between provinces. This results in big differences of inter-provincial industrial COD discharge intensity and its different spatial characteristics from industrial COD discharge amount (Figure 2b). In 2009, industrial COD discharge intensity was 187.04 tons/hundred million Yuan and 181.16 tons/hundred million Yuan in Ningxia Hui Autonomous Region and Guangxi Zhuang Autonomous Region, respectively, higher than that in other provinces; high in Xinjiang and Qinghai, 97.34 and 83.46, respectively; the lowest in Beijing, Shanghai and Tianjin, 2.13, 5.37 and 6.48, respectively; low in Guizhou, Guangdong, Fujian, Jiangsu and Shandong, between 10 and 20; and from 20 to 50 in other provinces.

The above analysis can only reflect the changing trend and *status quo* of Chinese inter-provincial industrial COD discharge amount and intensity in the past 15 years, but fails to reveal the evolution trends of industrial COD discharges in a spatial unit. 

### 3.2. Global Spatial Autocorrelation

**Figure 3:**
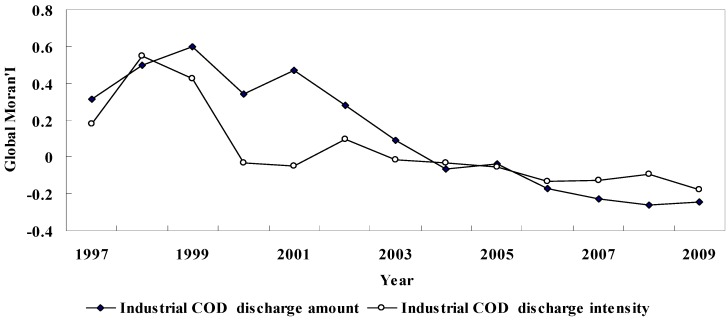
Global Moran’s I of industrial COD discharge amount and intensity.

Figure 3 is the Global Moran’s *I* values of industrial COD discharge amount and intensity in each year and different economic development stages. They give the spatial autocorrelation degree of these two indexes, and reflect their general changing trends in space.

Global Moran’s *I* of Chinese industrial COD discharge amount is generally on the downside, and in different economic development stages, with different characteristics (Figure 3). During the “9th Five-Year” period, Global Moran’s *I* value of industrial COD discharge amount took on the changing form of first ascending and then descending. It was 0.317 in 1997, increased to 0.579 in 1999 and later decreased to 0.342 in 2000, but despite the slight fluctuation of Global Moran’s *I*, Chinese industrial COD discharge amount was still represented by a strong positive spatial autocorrelation, displaying an agglomeration distribution pattern in the space. During the “10th Five-Year” period, Global Moran’s *I* value of industrial COD discharge amount showed a trend of notable decline, from 0.469 in 2001 to –0.036 in 2005. It transited from a positive spatial autocorrelation to uncorrelated, and displayed a conversion from an agglomeration pattern to a random distribution pattern. During the “11th Five-Year” period, Global Moran’s *I* value of industrial COD discharge amount maintained the downward tendency on the whole, from –0.173 in 2006 to –0.243 in 2009. There was a transition from uncorrelated at the end of the “10th Five-Year” period to a negative spatial autocorrelation, and in the space, from a random distribution pattern to a discretization pattern.

Global Moran’s *I* value of industrial COD discharge intensity is also on the downside like that of industrial COD discharge amount, but its variation range and characteristics in different economic development stages are not quite similar (Figure 3). During the “9th Five-Year” period, the Global Moran’s *I* curve of industrial COD discharge intensity assumed a “V” shape. It went from 0.181 in 1997 up to the highest value of 0.548 in 1998, and then down to −0.032 by 2000, *i.e.*, from weak to strong positive spatial autocorrelation and then to uncorrelated. As displayed in space, it was a conversion from an agglomeration pattern to a random distribution pattern. During the “10th Five-Year” period, the Global Moran’s *I* value of industrial COD discharge intensity was between −0.1 and 0.1, uncorrelated and represented by a random distribution pattern in the space. During the “11th Five-Year” period, the Global Moran’s *I* value of industrial COD discharge intensity was on the downside, down to −0.174 by 2009. It showed a weak negative spatial autocorrelation, demonstrating in space a variation trend from a random distribution pattern to an agglomeration pattern.

### 3.3. Local Spatial Autocorrelation

Global spatial autocorrelation enables us to judge the existence of spatial discretization or agglomeration phenomena, but fails to detect the location of agglomeration or discretization as well as the relation between spatial units. Local spatial autocorrelation makes up for this deficiency. It can reveal the similarity or correlation of attribution values between a spatial unit and its adjacent units, identify spatial agglomeration and spatial isolation, detect spatial heterogeneity, *etc.* To obtain a comparative analysis of variation laws of industrial COD discharge in space and at different times, this study calculates the industrial COD average discharge amount and intensity during the “9th Five-Year”, “10th Five-Year” and “11th Five-Year” periods, respectively, and uses LISA Agglomeration Diagram to study their spatial-temporal evolution.

**Figure 4:**
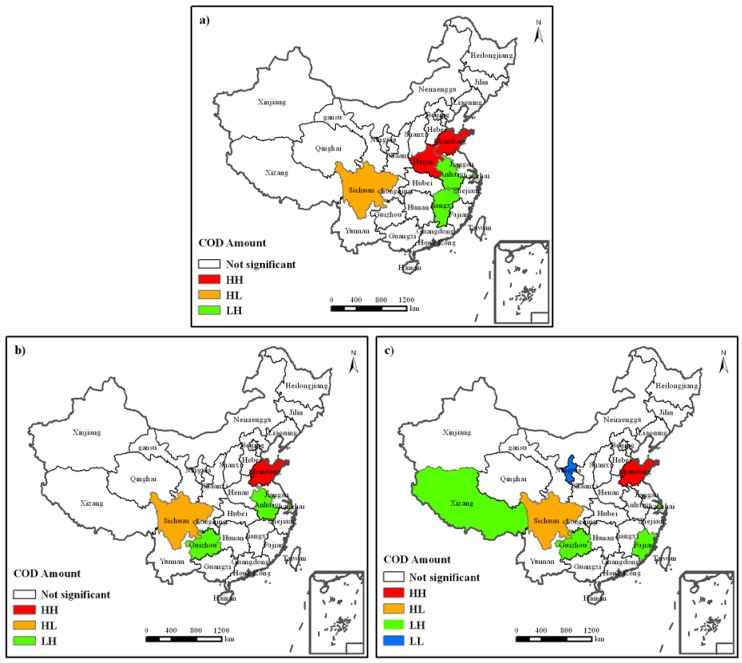
LISA cluster map of industrial COD discharge amount. Temporal and spatial variations of industrial COD discharge amount are divided into three phases. (**a**) Its status during the “9th Five-Year” period; (**b**) Its status during “10th Five-Year” period; and (**c**) Its status during “11th Five-Year” period. HH means High-High spatial pattern; HL means High-Low spatial pattern; LH means Low-High spatial pattern; LL means Low-Low spatial pattern.

Figure 4 gives the spatial pattern of industrial COD discharge amount during the “9th Five-Year” (Figure 4a), “10th Five-Year” (Figure 4b) and “11th Five-Year” (Figure 4c) periods, respectively. There is slight change on the whole. During the “9th Five-Year” period (Figure 4a), Shandong and Henan Provinces were located in High-High area of Local Moran’s *I*, being the high agglomeration area of industrial COD discharge amount; Sichuan Province in the High-Low area, indicating that it had high industrial COD discharge amount, but its adjacent provinces had a low one; Shanghai, Anhui and Jiangxi Provinces in the Low-High area, indicating that these three provinces had low industrial COD discharge amounts, and their adjacent provinces had high ones; other provinces had their industrial COD discharge amounts displayed in space as random distributions; and there is no agglomeration area of low value of industrial COD discharge amount. During the “10th Five-Year” period (Figure 4b), Shandong Province was the only one in the High-High area of Local Moran’s *I*, being a high agglomeration area of industrial COD discharge amount; Sichuan Province in the High-Low area, with the same spatial characteristics as during the “9th Five-Year” period; Shanghai, Anhui and Guizhou Provinces in the Low-High area; other provinces had their industrial COD discharge amounts displayed in space as a random distribution; and there was no agglomeration area of low value of industrial COD discharge amount, either. During the “11th Five-Year” period (Figure 4c), Shandong and Sichuan Provinces were located in the High-High and High-Low areas of Local Moran’s *I*, respectively, with the same spatial characteristics as during the “10^th^ Five-Year” period; The Low-High area where Shanghai, Guizhou, Fujian and Tibet were located displayed great changes; another important change was the location of the Ningxia Hui Autonomous Region in the Low-Low area, with a low value agglomeration of industrial COD discharge amount; other provinces had their industrial COD discharge amounts displayed in space as random distributions.

**Figure 5:**
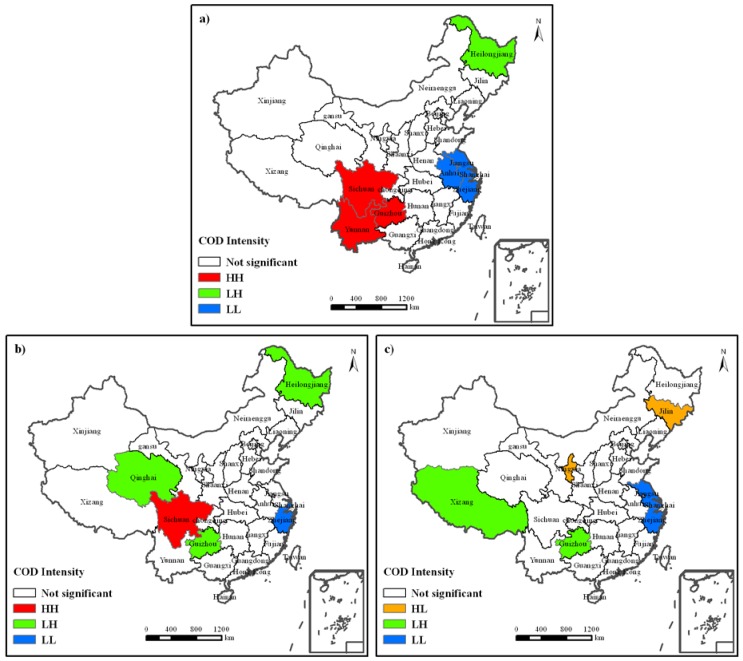
LISA cluster map of industrial COD discharge intensity. Temporal and spatial variations of industrial COD discharge amount are divided into three phases. (**a**) Its status during the “9th Five-Year” period; (**b**) Its status during the “10th Five-Year” period and (**c**) Its status during “11th Five-Year” period. The meaning of HH, HL, LH, LL is like those in Figure 4(a–c).

Figure 5 shows the spatial patterns of industrial COD discharge intensity during the “9th Five-Year” (Figure 5a), “10th Five-Year” (Figure 5b) and “11th Five-Year” (Figure 5c) periods, respectively. It demonstrates great changes in three different economic development stages. During the “9th Five-Year” period (Figure 5a), Sichuan, Guizhou and Yunnan Provinces were located in the High-High area, being the agglomeration area of high industrial COD discharge intensity; Heilongjiang Province was in the Low-High area, indicating that it had low industrial COD discharge intensity, and its adjacent provinces had relatively high ones; Jiangsu Province, Shanghai Municipality, Zhejiang Province and Anhui Province were in the Low-Low area, being low value agglomeration areas of industrial COD discharge intensity; there was no High-Low area; and other provinces had their industrial COD discharge intensity displayed as random distributions. During the “10th Five-Year” period (Figure 5b), there was a decrease in the high value agglomeration area of industrial COD discharge intensity, and Sichuan Province was the only province in the High-High area; there was no High-Low area; great changes occurred to the Low-High area, where Heilongjiang Province, Guizhou Province and Qinghai Province were located; there was a sharp cut in the Low-Low area, and Shanghai Municipality and Zhejiang Province were the low value agglomeration areas of industrial COD discharge intensity. During the “11th Five-Year” period (Figure 5c), the spatial pattern of industrial COD discharge intensity displayed two outstanding changes: first, there was no high value agglomeration area, *i.e.*, the High-High area disappeared; second was the emergence of High-Low areas in Jilin Province and the Ningxia Autonomous Region. Moreover, Jiangsu Province was added into the Low-Low area; and Tibet Autonomous Region and Guizhou Province became part of the Low-High area.

### 3.4. Reasons for Spatial Pattern of Industrial COD Discharge

Formation and evolution of industrial COD discharge spatial pattern depends on combination of social, economic, political and technological. Reduction of industrial COD discharge benefits from two aspects: stringent environmental regulations and increased funding for environmental protection. Firstly, along with economic development, Chinese government pays more attention to environmental pollution and concerns about environment protection.

In the southeast coast of China, an economical developed area, environment protection indexes including per unit GDP energy and discharge of major pollutants are listed first as governmental performance assessments. If an industrial enterprise is established in the industrial parks in Shanghai, Jiangsu and Zhejiang, its pollution control capacity is even regarded as one of the essential requirements. In 2012, discharge intensity is regarded as an evaluation target in the index system for achieving modernization established by the Jiangsu government. Guided by those polices, governments of economically underdeveloped areas pay more attention to pollutant reduction. Secondly, the Chinese government has increased funding for environmental protection. In these three economic development stages, funding for industrial wastewater treatment shows remarkable growth. During the “9th Five-Year” period, it was 32.29 billion Yuan, 47.11 billion Yuan during the “10th Five-Year” period, and it is up to 69.13 billion Yuan during the “11th Five-Year” period. Increased funding for environmental protection helps upgrade wastewater treatment facilities and improve treatment technology. The decrease of industrial COD discharge intensity is due to both the reduction of industrial COD discharge amounts and an annual increase of Chinese industrial output.

The spatial pattern of industrial COD discharge is determined by the regional differences of economic development, industrial scale, industrial pattern, industrial technology and environmental protection policies [36]. In China’s economic development programme, the Eastern coastal areas, especially the three big metropolitan areas, have obtained greater industrial development space and preferential policies than central and western areas do. This helps eastern areas realize the high speed economic development, which is obviously higher than that of central and western areas. Compared with the central and western areas, east China is provided with better location conditions, investment environment and economic basis, promoting the constant agglomeration of industry. Industrial agglomeration brings about technological competition between enterprises as well as technological upgrading and modification, thus advancing the saving and intensive utilization of resources. Moreover, in east China, much attention has been given to water pollution caused by industrialization, many funds have been input for pollution prevention and control, and stricter regulatory rules have been formulated for industrial pollution discharges. Consequently, the high value agglomeration areas of industrial COD discharge intensity appear in Sichuan, Guizhou, Yunnan and other western regions, while the low value agglomeration areas of industrial COD discharge intensity are in Jiangsu Province, Shanghai Municipality, Zhejiang Province and other eastern regions. For this reason, a differentiation strategy shall be adopted for Chinese industrial COD discharge reduction. Eastern regions shall increase the rate of cyclic utilization and cut down industrial COD discharge amounts through high efficiency integration of the industrial chain; central and western regions shall speed up the adjustment of industrial structure, strengthen the progress of industrial upgrading and modification, and adopt clean production technology to control the amount of industrial COD discharges.

Further quantitative research on the influences of economy, technology, policy, natural endowment and many other factors on industrial COD discharge patterns is required. Subject to the date restrictions, this study just conducts an analysis of the spatial evolution of industrial COD discharge amounts and intensity at the inter-provincial level. The time scale of the research presented herein is also short. In addition, despite the great importance of industrial COD discharge reduction, control of domestic COD discharges is also worthy of consideration. With the migration and agglomeration of population, the situation for supervision and control on domestic COD is getting worse and worse.

## 4. Conclusions

(1) In the last 15 years, the amount and intensity of industrial COD discharge are on a decrease, and the tendency is more remarkable for industrial COD discharge intensity. There are large differences between inter-provincial industrial COD discharge amounts and intensity, with different spatial differentiation features.
(2) Industrial COD discharge amount and intensity do not show strong spatial autocorrelation, but demonstrate agglomeration or convergence patterns in some certain years. Their Global Moran’s I is generally on the decrease. As displayed in space, there is also an evolution from an agglomeration pattern to a discretization pattern, but at different economic development stages, these two indexes have different Global Moran's I values and spatial distribution patterns; compared with industrial COD discharge amount, industrial COD discharge intensity has a weak spatial autocorrelation and it does not show any obvious spatial discretization or agglomeration patterns.
(3) Local spatial autocorrelation analysis shows that High-High areas of industrial COD discharge amount show small changes, and are mainly located in Shandong Province; High-Low areas have always been stable in Sichuan Province; Low-High and Low-Low areas vary largely, without specific regularity. The agglomeration area of industrial COD discharge intensity varies greatly in space with time, and High-High areas concentrate in the southwest in China, become smaller with time and disappeared during the “11th Five-Year” period; Low-Low areas mainly concentrate in Shanghai, Zhejiang and Jiangsu Provinces in the eastern coastal regions, and there is no regularity for High-Low and Low-High areas.
(4) Stringent environmental regulations and increased funding for environmental protections are the crucial factors to cut down industrial COD discharge amounts and intensity. Spatial patterns of industrial COD discharge are determined by the regional differences in economic development, industrial scale, industrial patterns, industrial technology and environmental protection policies.
